# Internet-Based Individualized Cognitive Behavioral Therapy for Shift Work Sleep Disorder Empowered by Well-Being Prediction: Protocol for a Pilot Study

**DOI:** 10.2196/24799

**Published:** 2021-03-18

**Authors:** Asami Ito-Masui, Eiji Kawamoto, Ryota Sakamoto, Han Yu, Akane Sano, Eishi Motomura, Hisashi Tanii, Shoko Sakano, Ryo Esumi, Hiroshi Imai, Motomu Shimaoka

**Affiliations:** 1 Departments of Molecular and Pathobiology and Cell Adhesion Biology Mie University Graduate School of Medicine Tsu City, Mie Japan; 2 Departments of Emergency and Disaster Medicine Mie University Graduate School of Medicine Tsu City, Mie Japan; 3 Emergency and Critical Care Center Mie University Hospital Tsu City, Mie Japan; 4 Department of Medical Informatics Mie University Hospital Tsu City, Mie Japan; 5 Department of Electrical and Computer Engineering Rice University Houston, TX United States; 6 Department of Neuropsychiatry Mie University Graduate School of Medicine Tsu City, Mie Japan; 7 Center for Physical and Mental Health Mie University Tsu City, Mie Japan; 8 Mie Prefectural Mental Medical Center Tsu City, Mie Japan

**Keywords:** shift work sleep disorders, health care workers, wearable sensors, shift work, sleep disorder, medical safety, safety issue, shift workers, sleep, safety, cognitive behavioral therapy, CBT, online intervention, pilot study, machine learning, well-being

## Abstract

**Background:**

Shift work sleep disorders (SWSDs) are associated with the high turnover rates of nurses, and are considered a major medical safety issue. However, initial management can be hampered by insufficient awareness. In recent years, it has become possible to visualize, collect, and analyze the work-life balance of health care workers with irregular sleeping and working habits using wearable sensors that can continuously monitor biometric data under real-life settings. In addition, internet-based cognitive behavioral therapy for psychiatric disorders has been shown to be effective. Application of wearable sensors and machine learning may potentially enhance the beneficial effects of internet-based cognitive behavioral therapy.

**Objective:**

In this study, we aim to develop and evaluate the effect of a new internet-based cognitive behavioral therapy for SWSD (iCBTS). This system includes current methods such as medical sleep advice, as well as machine learning well-being prediction to improve the sleep durations of shift workers and prevent declines in their well-being.

**Methods:**

This study consists of two phases: (1) preliminary data collection and machine learning for well-being prediction; (2) intervention and evaluation of iCBTS for SWSD. Shift workers in the intensive care unit at Mie University Hospital will wear a wearable sensor that collects biometric data and answer daily questionnaires regarding their well-being. They will subsequently be provided with an iCBTS app for 4 weeks. Sleep and well-being measurements between baseline and the intervention period will be compared.

**Results:**

Recruitment for phase 1 ended in October 2019. Recruitment for phase 2 has started in October 2020. Preliminary results are expected to be available by summer 2021.

**Conclusions:**

iCBTS empowered with well-being prediction is expected to improve the sleep durations of shift workers, thereby enhancing their overall well-being. Findings of this study will reveal the potential of this system for improving sleep disorders among shift workers.

**Trial Registration:**

UMIN Clinical Trials Registry UMIN000036122 (phase 1), UMIN000040547 (phase 2); https://tinyurl.com/dkfmmmje, https://upload.umin.ac.jp/cgi-open-bin/ctr_e/ctr_view.cgi?recptno=R000046284

**International Registered Report Identifier (IRRID):**

DERR1-10.2196/24799

## Introduction

### Shift Work Sleep Disorder and Burnout Syndrome

Long working hours and shortages of human resources among health care workers have increased the turnover rate among their ranks, which has become a major medical safety issue. Shift work sleep disorder (SWSD) is a circadian rhythm disorder presenting with excessive sleepiness or insomnia associated with shift work. The prevalence of SWSD varies among studies; however, evidence shows that more than one in five shift workers experience SWSD [1]. Irregular sleep among shift workers is recognized as a serious problem leading to burnout [2]. Medical workers in the field of emergency medicine and intensive care are at particularly high risk of burnout syndrome due to the high levels of physical and mental stress, along with irregular sleep and work schedules [3]. Early detection and improvement of conditions that may lead to burnout is a major issue worldwide.

Assessment of SWSD includes medical interviews, sleep diaries, and sleep self-reports. Motivation and adherence are central to SWSD management outcomes. Circadian adaptation with the help of bright light and melatonin treatment are part of the clinical treatment for SWSD. Another crucial component of treatment is improving sleep by practicing good sleep hygiene, adjusting sleep conditions, and taking effective naps. Many clinical approaches to SWSD can be considered broadly behavioral [1].

### Internet-Based Cognitive Behavioral Therapy and Machine Learning

Cognitive behavioral therapy (CBT) for insomnia (CBTI) is a nonpharmacological treatment option for insomnia. CBTI is a psychological approach for improving sleep by adjusting sleep-related habits and misconceptions regarding sleep, thereby reducing sleep-related problems. The main components of CBTI are sleep diaries, education on sleep hygiene, and counseling by sleep specialists. According to clinical practice guidelines for appropriate use and cessation of sleep medication published by the Japanese Society of Sleep Research [4], CBT is positioned as the second-line therapy when pharmacological treatment is unsuccessful. The guidelines further state current evidence showing that CBT is effective as first-line therapy or as combination therapy. With respect to treatment for SWSD, the Japanese Society of Neurological Therapeutics published treatment guidelines for insomnia, hypersomnia, and circadian rhythm disorder, stating that combination therapy of CBT with other treatments is a highly important option [5].

Studies have shown that smartphone apps and web-based internet-based CBTI are equally effective as face-to-face CBTI treatment [6,7]. Internet-based CBTI has been reported to improve not only sleep but also subjective well-being [8]. As discussed above, the behavioral therapy components for SWSD share some of the same components of CBTI; thus, we consider that similar methods can also be applied to SWSD. Since the research subjects of this study are not SWSD patients but rather healthy shift workers with a high risk of developing SWSD, we hypothesize that early intervention using this method will improve sleep and well-being in this population.

To further enhance the effect of CBT, we will incorporate machine learning–based well-being prediction. The roles of machine learning–based well-being prediction include increasing participants’ awareness of their well-being and behavior, and helping them to reflect and change their behavior (eg, sleep) to promote their well-being. If the results from the well-being prediction are shown to physicians, additional roles can include supporting physicians to review participants’ historical data (eg, Fitbit and surveys) along with behavioral and physiological changes, make clinical decisions, and provide advice before the conditions deteriorate.

### Wearable Sensors and Biometric Big Data

Evaluation and interventions for SWSD have been challenging owing to the lack of an objective measurement of sleep under irregular working conditions. Self-reported sleep hours are known to have low reliability and accuracy. However, the use of wearable sensors, which allow for real-time, continuous monitoring over long periods of time, has made it possible to visualize, collect, and analyze the work-life balance of health care workers experiencing irregular sleep and work conditions. A 7-day study of nurses using wearable sensors found that sleep duration, wake time, and napping correlated with fatigue [9]. In addition, the collection of biometric big data using wearable sensors has facilitated the application of machine learning associated with health care management strategies. Several studies have used these data to predict or detect certain states such as stress using machine learning [10]. Thus, applications of wearable sensors to evaluate sleep and work conditions in shift workers can provide important insights that may lead to improvement in quality of life.

### Objective

In this study, we hypothesize that internet-based CBT for SWSD (iCBTS), which implements machine learning–based well-being prediction, can improve the sleep durations of shift workers in both healthy and potential SWSD individuals, thereby preventing any decreases to their well-being. We aim to test this hypothesis by evaluating the effect of iCBTS on sleep duration and well-being using wearable sensors in a university hospital intensive care unit (ICU).

## Methods

### Study Overview

This is a prospective interventional pilot study consisting of two phases: (1) preliminary data collection and the development of machine-learning models for well-being prediction, and (2) intervention and evaluation of iCBTS for SWSD. The study will include shift workers (physicians and nurses) working at a university hospital ICU. Participants will be asked to wear a wrist-worn biometric tracker 24 hours a day and answer daily questionnaires about their well-being. After phase 1, sleep and well-being data will be used to build an algorithm for well-being prediction. Phase 2 begins with the baseline period (1 week; data collection only), followed by 4 weeks of the intervention period (data collection+intervention) (see details of the two periods in the following Measurements and Intervention subsections). We will compare sleep duration, well-being, and other variables during the baseline and intervention periods in all participants. The primary outcome of this study will be mean sleep duration at the last week of the intervention.

### Inclusion and Exclusion Criteria

Inclusion criteria are as follows: (1) shift workers at the Emergency and Critical Care Center, Mie University Hospital; (2) 8 hours of shift work per shift; (3) Pittsburgh Sleep Questionnaire (PSQI) score≥5. Written consent to participate in this study will be obtained after sufficient information is provided. Exclusion criteria are as follows: (1) diagnosis of sleep disorders such as sleep apnea, restless leg syndrome, and narcolepsy; (2) diagnosis of psychotic diseases such as depression, panic disorder, and anxiety disorder; (3) use of sleep medications; (4) pregnancy; (5) history of contact dermatitis or other skin diseases with a high risk for skin disorders; (6) patients who are judged by the principal investigator or subphysician to be unsuitable as research subjects.

### Measurements

For the collection of biometric data, we will use Fitbit Charge 3. Based on the primary data obtained by the wearable’s built-in accelerometer, altitude sensor, and heart rate sensor, this wearable sensor allows for the monitoring and collection of the following biometric information: sleep-related information (start/end time of sleep, minutes awake during sleep, time of each sleep stage), heart rate, and information on activity (number of steps, calories burned, intensity of exercise). Personal information other than the above (eg, location information, phone numbers) cannot be accessed.

For subjective assessment of well-being, participants will complete morning and evening daily surveys distributed via email or the app. Subjective well-being consists of five categories: alertness, happiness, energy, health, and calmness. The participants will self-evaluate each category with a score ranging from 0 to 100. Other questions in the survey include subjective sleep evaluation (eg, sleepiness, awakenings, satisfaction), and daily activities and habits (eg, events, alcohol and caffeine intake). In addition, as an objective measurement of response to stimuli, participants will take the 3-minute Psychomotor Vigilance Test along with the survey [11]. For evaluation of baseline characteristics and sleep-related conditions, participants will take the following questionnaires before and after intervention: the PSQI, Japanese Epworth Sleepiness Scale (JESS), General Health Questionnaire (GHQ), and State-Trait Anxiety Inventory (STAI).

During the preintervention period, participants are prohibited to access their own activity and sleep data; however, during the intervention period, participants are free to access their own activity and sleep data, and are given personalized sleep advice from physicians 4 times per week (intervention week 1) followed by 3 times per week (intervention weeks 2-4).

### Intervention

**Figure 1:**
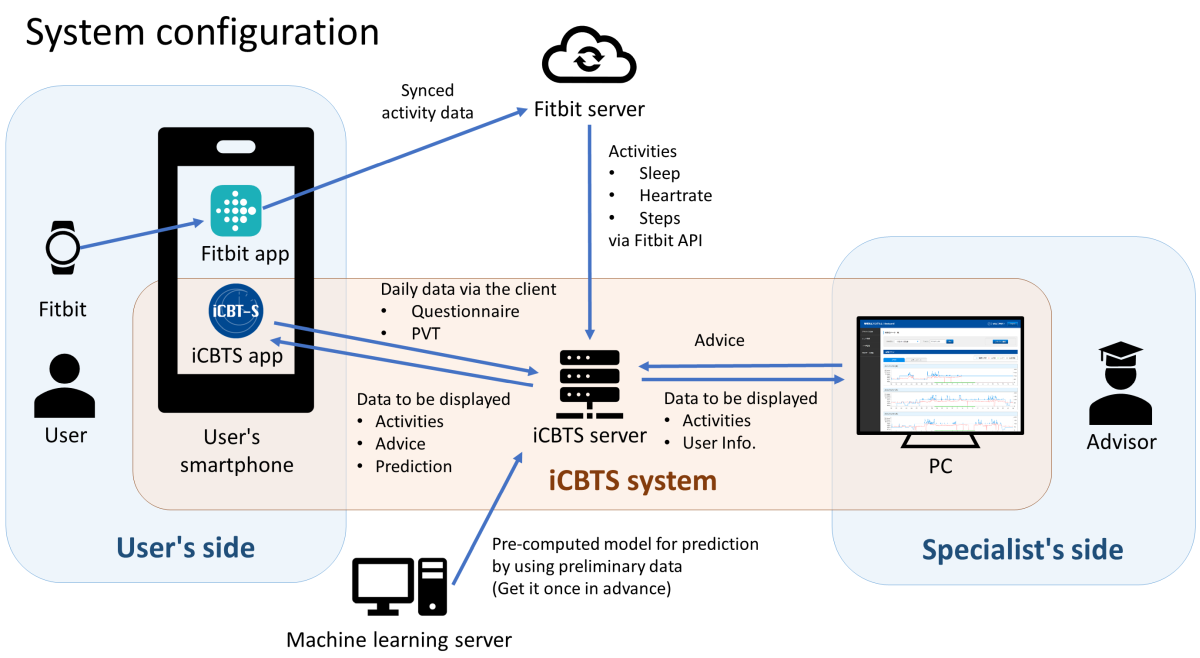
The internet-based cognitive behavioral therapy for shift work sleep disorder (iCBTS) system uses a smartphone app as a client to obtain questionnaires and other manual entry information from users. The system also provides a management portal for sleep professionals. Advice entered by specialists and predictions based on daily activities from Fitbit are displayed in the user's app.

The smartphone app for the intervention will be developed and available as a Progressive Web Application. No installation is required, and the app can be used on an iOS or Android device. Once the shortcut is placed on the user’s home screen, they can immediately access all of the app services. To sign in, participants must enter an ID and password that will be assigned to each individual. The smartphone app is a client of the iCBTS system and is responsible for collecting manual entry information from users, such as questionnaires. The system also provides research coordinators and sleep specialists with a management portal for writing and sending advice to users. The system can further predict well-being based on the model obtained in advance along with daily data collected from Fitbit, and feeds back to the user’s app with advice (Figure 1). The dashboard contains three main features: well-being prediction of the day, the surveys that need to be answered, and the shift schedule of the participant.

Well-being prediction is one of the essential components of the app. Based on earlier studies of machine learning and subjective well-being [12,13], we have categorized well-being into five components: alertness, happiness, energy, physical health, and calmness. These prior studies have predicted well-being labels using wearable sensors, mobile phones, and surveys. These studies have used the following metrics to evaluate the well-being prediction performance: accuracy in classification and mean absolute error (MAE) in regression models, calculated as follows:

Accuracy: Number of true predictions/(number of true predictions + number of false predictions)

MAE: mean (|ground truth – predicted value|)

Yu et al [13] used passive mobile phone data (eg, phone calls, SMS text messages, screen usage) and wearable sensor data (electrodermal activity, skin temperature, body accelerometer) from 243 college students to develop a long short-time transfer learning neural network model that could predict the students’ next-day well-being status, including subjective happiness, physical health, and calmness, in a 0-100 regression task. The MAE of the regression models were 13.5, 13.2, and 14.4 for happiness, health, and calmness, respectively. Yu et al [14] also leveraged wearable sensor data (eg, heart rate, step count, sleep) and questionnaire responses (eg, work hours, caffeine/alcohol/drug intake) from 14 physicians and nurses. They built a job role–based well-being prediction model for predicting the next days’ self-reported alertness, happiness, energy, health, and calmness with model performances of 64%, 79%, 71%, 81%, and 84%, respectively, in high/low binary classification; 59%, 52%, 51%, 58%, and 57%, respectively, in high/mid/low three-class classification; and 17.4, 15.1, 17.7, 15.4, and 15.6, respectively in regression.

**Figure 2:**
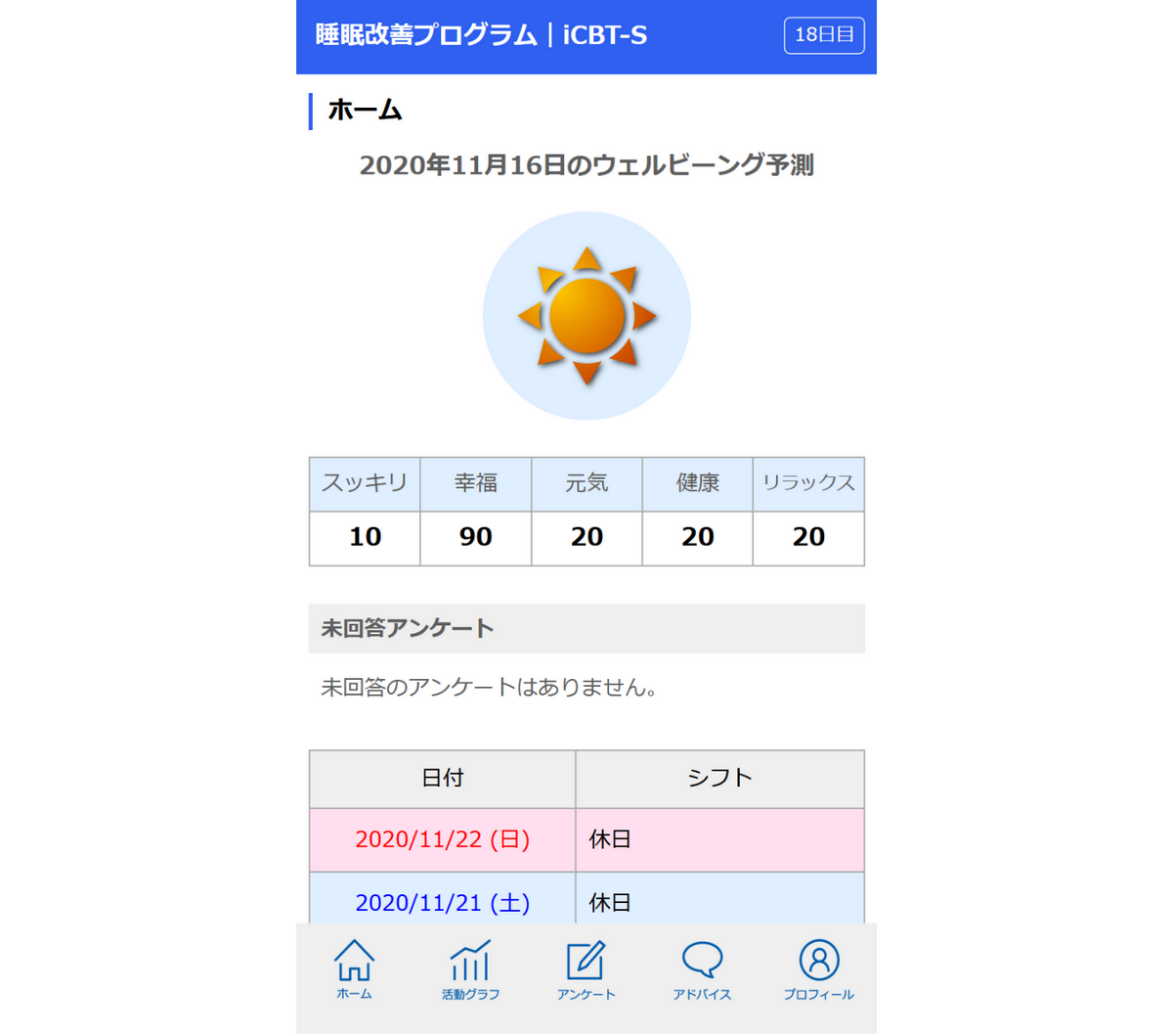
Well-being prediction is displayed in numbers (rounded to the nearest 10) and as a visual icon that resembles a weather forecast: cloudy for scores 0-50, cloudy and partly sunny for scores 60-80, and sunny for scores 90-100.

In this study, well-being prediction will be based on these five self-reported labels on a scale of 0 to 100, which are collected twice daily (8 AM and 8 PM). To predict well-being scores, we developed a job-based multitask and multilabel convolutional neural network–based well-being prediction model using pilot data from 14 participants with a total of 241 days of data collection [14]. Twenty-three daily features were extracted from the wearable sensor and daily surveys, including biobehavioral features such as sleep duration, sleep variability, the number of steps, and average and variability of heart rate for the previous 1-7 days; work-related features such as shift schedule and working hours; and daily habits such as alcohol and caffeine intake. The model extracted high-level features using convolutional kernels and predicted five well-being scores simultaneously for physicians and nurses. The prediction performance of the proposed model was compared with that of control models, including job role–based multitask only and multilabel only (doctors only, nurses only, and all participants), and baseline models such as support vector machine and random forest. The proposed model achieved the best performance in almost all of the evaluations performed. In particular, the happiness score, which is considered to be representative of well-being, is illustrated by a graphic that resembles a weather forecast: cloudy for scores 0-50, cloudy and partly sunny for scores 60-80, and sunny for scores 90-100 (Figure 2). The well-being prediction result is updated every morning on the app screen based on the data collected up to the previous evening.

**Figure 3:**
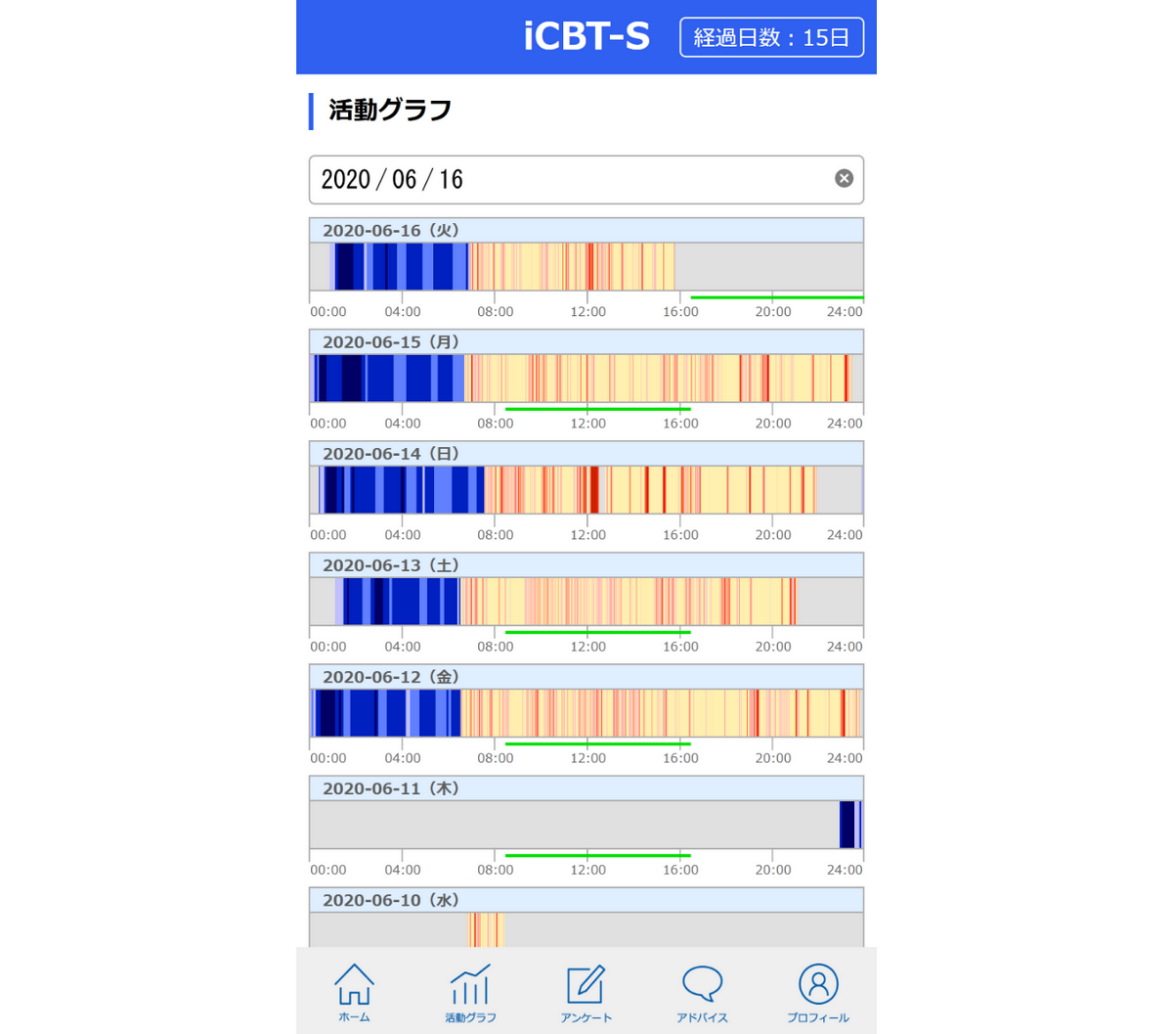
The app has a total of five tabs: “dashboard,” “activity/sleep chart,” “surveys,” “advice,” and “user profile.” On the “activity/sleep chart” tab, participants will see at a glance how much activity and sleep they have had in 24 hours for the past 7 days.

The app has a total of five tabs: “dashboard,” “activity/sleep chart,” “surveys,” “advice,” and “user profile.” On the “activity/sleep chart” tab, participants will see at a glance how much activity and sleep they have had in 24 hours for the past 7 days. These data are retrieved every hour from the Fitbit server using its web application programming interface. Awakenings are displayed as red bars, where higher activity levels (higher number of steps per minute) are shown in deeper shades of red, and sleep episodes are displayed as blue bars, where deeper sleep stages are shown in deeper shades of blue (Figure 3).

On the “advice” tab, participants read personalized sleep advice from physicians who specialize in sleep, which are distributed 3 to 4 times a week. The physicians are anonymous to the participants, providing only information on their age, sex, and profession as their profile. Physicians conduct their assessments based on the 23 daily features collected via wearable sensor and surveys. Well-being prediction results are not shown to the physicians; thus, the sleep advice is based solely on clinical judgment of the physicians. Physicians will choose 3-5 messages among 23 fixed-format sleep advice messages (eg, “Avoid alcohol before you go to sleep,” “Try to wake up at a consistent time”). Participants are notified as soon as the sleep advice is written and sent. They are also asked to send their response indicating whether or not they are eager to comply with each piece of advice, in which a thumbs up indicates “eager to follow” and a thumbs down indicates “difficult to follow.” However, the physicians do not further reply to the comments. The aim for this feedback is to ensure that participants read the advice in a timely fashion, and to allow for their interactive participation, thereby potentially enhancing their motivation to continue.

During the baseline data collection period, participants are unable to access most of the features of the app and can only enter the surveys. After day 8 of the study, they will gain access to all of the app features.

### Sample Size and Power Calculation

The results of the study performed during phase 1 showed that the mean sleep duration among 16 shift workers was 334.68 (SD 135.1) minutes. The mean sleep duration in the baseline period of phase 2 is assumed to be statistically similar to that assessed in phase 1, as these phases are performed under similar conditions. Based on the pilot study data with a small sample, mean sleep duration at week 4 of the intervention was assumed to be 30 minutes longer, with no change in the SD. Assuming a two-sided level of significance of 5%, power of 80%, and correlation coefficient of 0.8, using the SAS system (SAS Institute Inc, Cary, NC), the number of participants needed for a significant difference to be found in the paired *t* test was calculated to be 66. We set the required number of participants at 70 because the dropout rate of the study was assumed to be about 5% of the enrolled individuals. Dropout rates of face-to-face CBTI in randomized controlled trials are reported to range from 0% to 8% [15]. According to prior studies, the mean attrition rate of internet-based psychological intervention programs performed in the workplace is 23%, with a range of 3% to 54% [16]. Dropout rates of internet-based sessions tend to be higher than those of face-to-face sessions. However, we estimate that a low dropout rate can be achieved due to the high frequency of feedback from sleep physicians. In addition, a prior study performed in the same ICU targeting health care workers using wearable sensors showed a low dropout rate of 0% [17]. At Mie University Hospital where this study is performed, approximately 75 medical staff members are working in the ICU at any given time, and about 95% of them can be expected to provide consent and participate in the study.

### Outcome and Statistical Analysis

The primary outcome of this study is mean sleep duration on week 4 of the intervention. The means and SDs of sleep duration will be calculated at both the baseline data collection period and at week 4 of the intervention period. For the primary endpoint, a two-sided paired *t* test will be performed and assessed at the 5% significance level. In addition, the mean and SD of the changes in sleep duration will be calculated based on the difference between the mean sleep duration of the baseline period and week 4 of the intervention period.

The secondary outcomes of this study are as follows: activity (calories burned, steps taken), subjective well-being, reaction time, and quality and quantity of sleep. Median and quartile values for week 1, week 4, and for the rate of change will be calculated, and the Wilcoxon signed-rank test will be performed at the 5% significance level on both sides. In addition, we will perform a regression analysis of the time-series data (autoregressive model). This analysis will include all data collected during the study period as opposed to other analyses, which will use only data collected at the first and last weeks of the study. The median and quartiles will be calculated for the PSQI, JESS, STAI, and GHQ scores at both pretest and posttest, and the Wilcoxon signed-rank test will be performed at the 5% significance level on both sides. Using the cases in phase 1 as the control group and the cases in phase 2 as the intervention group, the means and SDs of the change in sleep duration will be calculated, and the Student *t* test will be performed at a significance level of 5% on both sides. Additionally, descriptive analysis will be performed to summarize the baseline characteristics of the participants in both groups.

## Results

This study was reviewed by The Clinical Research Ethics Review Committee of Mie University Hospital and was approved on January 25, 2019 (phase1) and May 17, 2020 (phase 2). Both trials are registered with the UMIN Clinical Trials Registry (phase 1: UMIN000036122, phase 2: UMIN000040547). Data collection in phase 1 was completed in October 2019, with full data obtained from 16 participants. In phase 1, the mean sleep duration was 334.68 (SD 135.1) minutes. As of September 2020, additional results from the trial were still being analyzed and evaluated. After revising and validating the optimal app function, recruitment for phase 2 started in October 2020. This study is expected to be completed by summer 2021.

## Discussion

The goal of this study is to explore the efficacy of iCBTS empowered by machine learning well-being prediction to improve the sleep durations and well-being of medical shift workers. Several insights could be derived from this study. First, we expect to clarify the effect of iCBTS for improving sleep and well-being in healthy shift workers, thus indicating internet-based CBT as a potentially effective preventive method for SWSD. Second, we will determine the role of machine learning in CBT. In a broad sense, machine learning in the field of medicine is rapidly expanding, particularly in areas such as risk stratification [18], medical imaging [19], and clinical diagnosis [20]. One of the purposes of CBT is to prompt patients to change the way they think or notice their own misconceptions. We propose that well-being prediction may prompt the user to become more aware of their well-being and behaviors that may influence their well-being, which may in turn promote behavioral change.

The technological limitations of this study are the sensing accuracy of the wearable sensor, which can be mediated by several factors under real-life settings. Even though several validation studies have already compared wearable sensor technology to conventional monitoring methods, and have indeed showed promising results [21], no scientific consensus has been reached to date. A second limitation is the difficulty of tracking subjective well-being, which can likely vary considerably throughout the day. Although increasing the number of sampling self-reported surveys may alleviate this problem, we believe that a higher frequency of self-reported well-being sampling to be challenging for busy shift workers. Another potential limitation is the accuracy of well-being prediction, which is difficult to validate. Moreover, this study cannot determine whether iCBTS is effective above mere participation in the study.

Future goals of this study include improvement and adjustment of well-being prediction algorithms, and strategies for the effective use of this iCBTS method to better support medical managers and physicians. In addition, further research is needed to examine the results in different organizational settings with shift workers.

## Abbreviations

CBT: cognitive behavioral therapy
CBTI: cognitive behavioral therapy for insomnia
GHQ: General Health Questionnaire
iCBTS: internet-based cognitive behavioral therapy for shift work sleep disorder
ICU: intensive care unit
JESS: Japanese Epworth Sleepiness Scale
MAE: mean absolute error
PSQI: Pittsburgh Sleep Questionnaire
STAI: State-Trait Anxiety Inventory
SWSD: shift work sleep disorder
